# Susceptibility of outer hair cells to cholesterol chelator 2-hydroxypropyl-β-cyclodextrine is prestin-dependent

**DOI:** 10.1038/srep21973

**Published:** 2016-02-23

**Authors:** Satoe Takahashi, Kazuaki Homma, Yingjie Zhou, Shinichi Nishimura, Chongwen Duan, Jessie Chen, Aisha Ahmad, Mary Ann Cheatham, Jing Zheng

**Affiliations:** 1 Department of Otolaryngology – Head and Neck Surgery, Feinberg School of Medicine, Northwestern University, Chicago IL 60611, USA; 2 Department of Communication Sciences and Disorders, Northwestern University, Evanston, IL 60208, USA; 3 Knowles Hearing Center, Northwestern University, Evanston, IL 60208, USA; 4 Division of Bioinformatics and Chemical Genomics, Graduate School of Pharmaceutical Sciences, Kyoto University, Kyoto, 606-8501, Japan; 5 Chemical Genomics Research Group, RIKEN Center for Sustainable Resource Science, Wako, Saitama 351-0198, Japan

## Abstract

Niemann-Pick type C1 disease (NPC1) is a fatal genetic disorder caused by impaired intracellular cholesterol trafficking. Recent studies reported ototoxicity of 2-hydroxypropyl- β-cyclodextrin (HPβCD), a cholesterol chelator and the only promising treatment for NPC1. Because outer hair cells (OHCs) are the only cochlear cells affected by HPβCD, we investigated whether prestin, an OHC-specific motor protein, might be involved. Single, high-dose administration of HPβCD resulted in OHC death in prestin wildtype (WT) mice whereas OHCs were largely spared in prestin knockout (KO) mice in the basal region, implicating prestin’s involvement in ototoxicity of HPβCD. We found that prestin can interact with cholesterol *in vitro*, suggesting that HPβCD-induced ototoxicity may involve disruption of this interaction. Time-lapse analysis revealed that OHCs isolated from WT animals rapidly deteriorated upon HPβCD treatment while those from prestin-KOs tolerated the same regimen. These results suggest that a prestin-dependent mechanism contributes to HPβCD ototoxicity.

Cholesterol is an essential component of the eukaryotic plasma membrane, which plays important roles in many physiological processes. Alterations of cholesterol levels can modify physical properties of the cell membrane such as thickness and fluidity, thereby affecting various membrane proteins^1^. In addition, direct binding of cholesterol to membrane proteins is reported to have functional importance^2^. Signal transduction through lipid-raft formation also depends on the cholesterol content of the membrane^3^. One of the commonly used methods for membrane cholesterol manipulation is the use of cyclodextrins (CDs)^4^, a family of cyclic oligosaccharides consisting of α-D-glucopyranose molecules joined in a ring form. CDs can complex with hydrophobic molecules such as cholesterol through their hydrophobic core and enhance their solubility via their hydrophilic outer surface. Because of this amphipathic nature, CDs are widely used in a variety of applications in the food and pharmaceutical industries from mobilization of cholesterol to drug delivery^5^.

Recently, 2-hydroxypropyl-β-cyclodextrin (HPβCD) has attracted particular interest, as it was demonstrated to alleviate the cholesterol transport deficiency in cells isolated from Niemann-Pick disease type C1 (NPC1) patients^6^. NPC1 is a rare genetic disorder characterized by a failure to traffic intracellular cholesterol and lipids, causing progressive neural degeneration and early death in affected individuals^7^. Although studies in NPC1 animal models established the effectiveness of HPβCD^8^,^9^, the compound is unfortunately ototoxic^10^ even in human NPC1 patients^11^,^12^. Recent studies have reported that HPβCD-treated mouse cochleae exhibited loss of outer hair cells (OHCs), but not other cells in the organ of Corti (OC) or in other major organ systems regardless of the mode of administration^10^,^13^. These observations suggest that OHCs are particularly vulnerable to cholesterol extraction by HPβCD, and that an OHC-specific mechanism may underlie the ototoxicity.

OHCs are sensory cells specialized for cochlear amplification, which mediates sensitivity and sharp frequency selectivity in mammals^14^. Prestin, an OHC-specific molecular motor protein, is required for this active mechanical amplification function^15^,^16^. Although prestin belongs to a diverse anion transporter family, solute carrier protein 26 (SLC26), it (SLC26A5) is the only member that confers so-called electromotility on cells^17^. It is estimated that 60–75% of the OHC’s lateral wall surface area is packed with ~11 nm particles, which are presumed to be comprised of prestin tetramers^18^,^19^. As a membrane protein, prestin’s function is significantly modulated by cholesterol manipulation^20^,^21^,^22^. It is generally thought that cholesterol’s effect on prestin is indirect and occurs by altering the physical properties of the lateral membrane (LM) where prestin resides, similar to numerous factors that change lipid membrane properties including membrane tension, temperature, and lipid thickness^23^,^24^,^25^,^26^,^27^. However, it is not clear why OHCs are more vulnerable to HPβCD as compared to other cells in the OC.

In this study, we sought to understand critical determinants that mediate OHC death caused by HPβCD treatment. We identified prestin, an OHC-specific motor protein, as an important factor that influences OHC fate in response to HPβCD. Using an *in vitro* system, we determined that prestin is capable of binding to cholesterol, which implies that cholesterol depletion by HPβCD may interfere with this interaction. Consistent with this idea, application of HPβCD on isolated OHCs or OCs resulted in rapid cell shrinking and occasional rupture, indicating that the membrane is compromised. Interestingly, sense organs from prestin-KO mice resisted the same HPβCD treatment. Taken together, our results underscore the potential contribution of prestin to the toxic effects of cholesterol depletion, which needs to be considered when employing combinatorial therapeutic strategies using HPβCD in NPC1 patients.

## Results

### Prestin-dependent susceptibility of OHCs to HPβCD treatment

**Figure 1:**
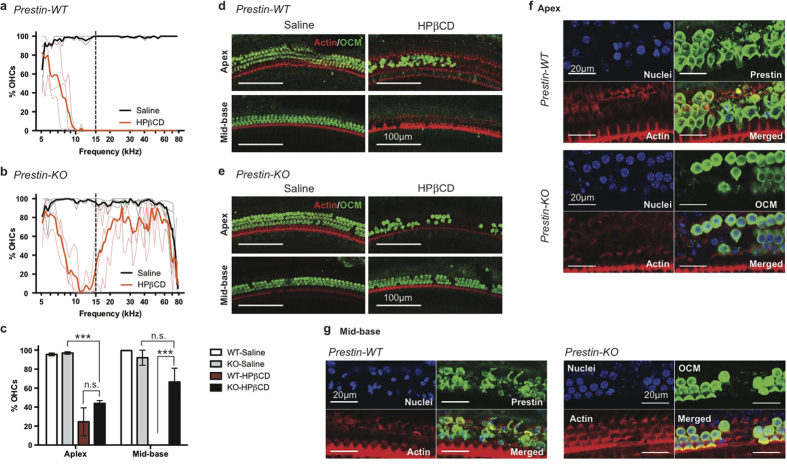
OHC loss in WT exceeds that in prestin-KO mice after HPβCD treatment. (**a,b**) WT and prestin-KO mice from a het x het breeder pair on the FVB background were treated with either saline or 8000 mg/kg HPβCD at P20–21. OHC survival was determined 7 days post injection. Thin lines represent the results from individual animals while thick lines are the average of each treatment (WT saline, n = 2; WT HPβCD n = 3; KO saline, n = 3; HPβCD, n = 3). OHC loss in the mid-to-basal region is observed in all WT cochleae, while OHCs were better preserved in KO cochleae. The dashed line indicates the boundary between apex and mid-base used in (**c**–**e**). (**c**) OHC survival is significant in mid-to-basal regions of HPβCD-treated prestin-KO cochlea. Mean ± SD are plotted, and significance determined using one-way ANOVA with Tukey’s post analysis. n.s., not significant; ***p ≤* 0.001*. (**d,e**) Representative images of saline or HPβCD-treated cochleae from WT (**d**) and prestin-KO (**e**) animals at apex and mid-base. Anti-oncomodulin (OCM) antibody was used to stain OHCs, and phalloidin-Alexa 546 for actin. Scale bars, 100 μm. (**f,g**) WT mice on the FVB background were treated with 8000 mg/kg HPβCD at P21 for 8 hours, and their cochleae harvested for immunofluorescence. The whole mount OC sections were stained with anti-prestin (WT) or anti-oncomodulin (KO) antibodies, Phalloidin-Alexa 546, and Hoechst 33342 for OHCs, actin, and nuclei, respectively. (**f**) Representative images of HPβCD-treated cochleae from WT (top panels) and prestin-KO (bottom panels) at the apex. Early events of OHC loss were observed for both WT and KO. For WT, fragmented OHCs were seen (judged by smooth vs. punctate prestin staining). For prestin-KO, absence of OCM staining indicates OHC loss. (**g**) Representative images of HPβCD-treated cochleae from prestin-WT and KO at the mid-base region of the cochlea. No intact OHCs were present in WT (left panels), although condensed nuclei indicating dying cells were observed. OHCs are largely intact in KO mice (right panels). Scale bars, 20 μm.

Since OHCs were the only cells lost after HPβCD treatment^10^, and since cholesterol manipulation modulates the function of the OHC-specific protein, prestin^21^, we reasoned that prestin may be involved in HPβCD-induced OHC loss. WT and prestin-KO littermates^28^ were, therefore, treated in parallel with the high dose of HPβCD (8000 mg/kg) previously shown to cause hearing loss^10^. We confirmed that HPβCD-treated WT cochleae examined 7 days post treatment exhibited massive OHC loss (Fig. 1a). Interestingly, HPβCD treatment of prestin-KO mice resulted in a similar loss of OHCs in the apical region but OHCs in the mid-to-basal regions were largely preserved (Fig. 1b,c). Inner hair cells (IHCs) and other supporting cell types in the OC were unaffected by the treatment in all the animals tested, confirming that the toxic effect of HPβCD is limited to OHCs as previously reported^10^ (Supplemental Fig. 1). Representative images in Fig. 1d,e highlight the differential toxicity of HPβCD between WT and prestin-KO mice, which is independent of genetic background. Similar patterns of OHC loss/preservation were observed in both FVB and 129/B6 strains (WT n = 10 and KO n = 8). Closer examination of HPβCD-treated OCs from WT animals revealed condensed nuclear staining and fragmented membrane staining in WT OHCs as early as at 8 hours post injection (Fig. 1f,g). Some OHC loss was also evident in prestin-KO cochleae at this time, indicating that the action of HPβCD is rather fast (Fig. 1f,g).

**Figure 2:**
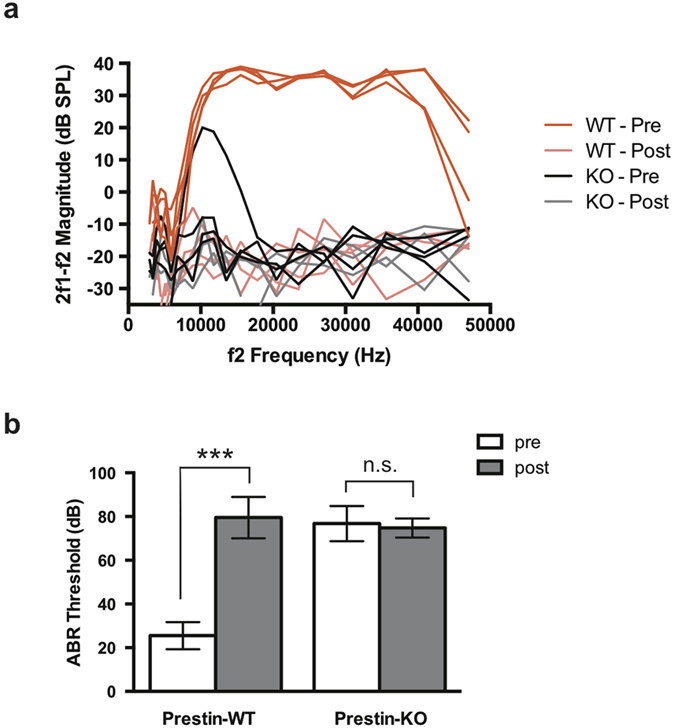
*In vivo* physiology of HPβCD-treated WT- and prestin-KO mice. (**a**) Distortion product otoacoustic emissions (DPOAEs) for WT and prestin-KO mice (n = 4 each) on the FVB background were recorded for L1 = L2 = 70 dB SPL before (“Pre”) and 7 days after (“Post”) 8000 mg/kg HPβCD treatment as in Fig. 1. HPβCD treatment abolishes DPOAEs in WT mice (faint red traces). Because prestin-KO mice (black traces) already have reduced emissions (Liberman *et al*., 2002), HPβCD did not affect DPOAEs in these mice as dramatically as in controls. (**b**) Auditory brainstem responses (ABR) from the same WT and prestin-KO mice as in (**a**) were determined using a 32 kHz tone burst before (“Pre”) and after (“Post”) HPβCD treatment. HPβCD treatment significantly increased the ABR threshold in WT mice. Prestin-KO mice already have a large threshold shift (Liberman *et al*., 2002) so that HPβCD did not affect ABR thresholds in these mice. Mean ± SD are plotted; significance was determined using an unpaired t-test. n.s., not significant; ***p ≤* 0.0001.*

We also measured cochlear function before and 7–8 days post treatment. As expected, both distortion product otoacoustic emissions (DPOAE) and auditory brain stem responses (ABR) were significantly impaired by HPβCD treatment in WT mice. *In vivo* physiological measurements showed loss of DPOAEs for equal level primaries at 70 dB SPL at all f2 frequencies (Fig. 2a) and increased ABR thresholds at 32 kHz (Fig. 2b) on the order of 40–50 dB. This change in phenotype is consistent with loss of OHC function. Anatomical examination of the HPβCD-treated WT mice showed massive OHC loss, but other cell types including IHCs and supporting cells remained intact (Fig. 1 and Supplemental Fig. 1). Because untreated prestin-KO mice lack OHC electromotility and show large reductions in neural responses^28^,^29^, HPβCD treatment had a minimal effect on DPOAEs and ABRs in mice lacking prestin (Fig. 2a,b) due to the loss of cochlear amplification. Only in one prestin-KO were DPOAEs measured at 70 dB and these responses for low f2 frequencies were lost after treatment. The observation that WT mice after HPβCD injection exhibited ABR thresholds similar to prestin KOs before treatment is consistent with the anatomical data showing preservation of IHCs and supporting cells in WT controls following cholesterol manipulation. If IHCs and/or spiral ganglion neurons had also been severely affected, a further loss of sensitivity would have been anticipated. Taken together, the data suggest that prestin is one of the mediators of OHC loss caused by HPβCD treatment.

### Prestin-dependent distribution of cholesterol in the OHC membrane

**Figure 3:**
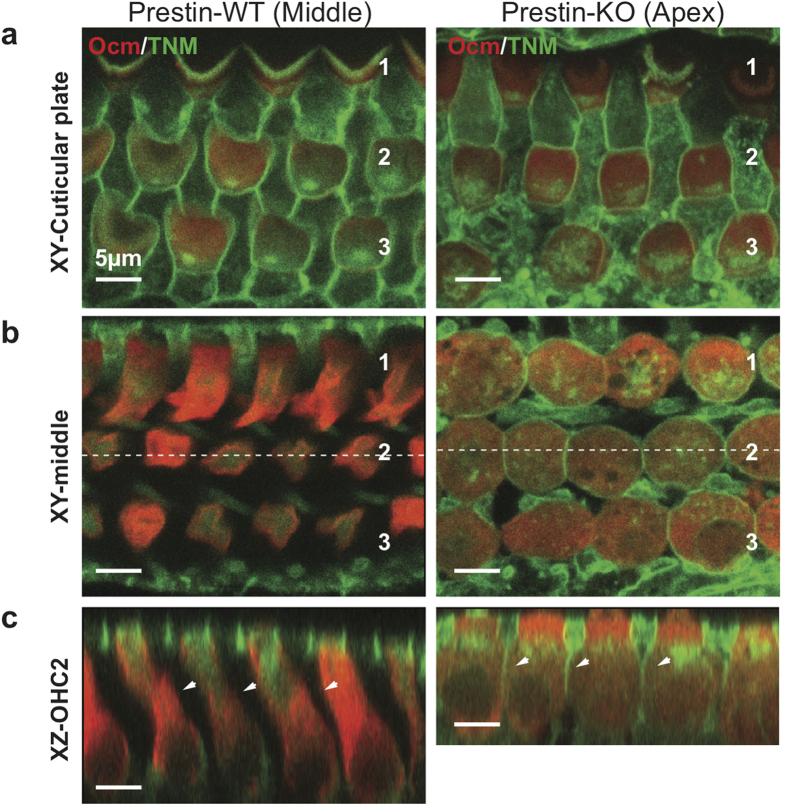
Cholesterol staining of cochleae from WT- and prestin-KO mice. Saline-treated WT- and prestin-KO cochleae (as in Fig. 1) were stained with TNM-fluorescein (TNM-FL) and imaged. (**a**) Single plane image at the cuticular plate (XY view, “Cuticular plate”) and (**b)** along the lateral wall (XY view, “middle”). Both WT and KO cochleae show similar cholesterol staining (green) for all rows of OHCs at the cuticular plate. (**c**) Radial view of the z-stack images. Unlike the KO, staining was not observed on the lateral walls of OHCs from WT cochlea (XZ view, “OHC-2”, white arrowheads). Red, anti-oncomodulin; Green, TNM-FL. Scale bars, 5 μm.

β−cyclodextrins are known to extract cholesterol from the plasma membrane^30^,^31^. Since OHCs from prestin-KO animals tolerated HPβCD treatment (Fig. 1), we wondered whether cholesterol distribution differed between OHCs with or without prestin. The cholesterol content of the OHC’s lateral membrane (LM) is low based on filipin staining, a polyene antifungal that binds to a 3β-hydroxyl group of cholesterol^4^,^20^,^21^,^32^. Because of its fluorescent nature, filipin is commonly used to detect cholesterol. However, it is not very stable and photobleaches rapidly. In order to reevaluate the cholesterol distribution in the inner ear with and without prestin, we utilized a cholesterol-binding small molecule, theonellamide (TNM). TNM was originally isolated from a marine sponge, and has been reported to bind in a specific manner^33^,^34^ to the same 3β-hydroxyl group of cholesterol as does filipin. Staining patterns in WT mice using fluorescein-conjugated TNM (TNM-FL)^35^ were consistent with previous observations using filipin: high levels of staining are seen at the apical and basal regions of each OHC, while the lateral walls where prestin resides are not stained regardless of the position of OHCs within the cochlea (Fig. 3a,b). Although OHCs in prestin-KOs are ~40% shorter than WT^28^,^36^,^37^, the TNM-FL staining revealed that cholesterol was present in the LM as well as in apical and basal regions (Fig. 3c). This cholesterol-staining pattern in mice lacking prestin is evident in the apical half of cochlea, but not in the base probably because the cells become short, which reduces the LM dramatically. In contrast to WT OHCs, other polarized cells in the OC, such as IHCs and supporting cells, are also evenly stained by TNM-FL regardless of position (Supplemental Figs 1 and 2). No noticeable reduction of TNM-FL staining was observed 7 days post treatment independent of whether prestin was expressed or not^10^ (Supplemental Fig. 2). Thus, OHCs that express prestin have an uneven cholesterol distribution, which appears to be absent in OHCs that lack prestin and in other cell types in the OC, suggesting a unique relationship between prestin and cholesterol.

### Interaction of prestin and cholesterol

Prestin is essential for OHC electromotility, structural integrity, and survival^15^,^28^,^37^,^38^. Previous studies have described a variety of cholesterol effects on prestin regulation, including its membrane targeting, oligomerization, and lipid-raft association^21^,^39^,^40^, as well as its voltage-dependent motor function^20^,^41^. These effects can be attributed to changes in physical properties of the biological membrane and/or to direct functional modulation through specific binding^41^. Although OHCs are highly susceptible to HPβCD, filipin and TMN did not stain the prestin-containing LM of OHCs. This observation raises the possibility that filipin and TMN may not access cholesterol when it is already in a complex with prestin and/or other proteins in the LM. In fact, it has been reported that some cholesterol-containing membranes are not labeled by filipin^42^,^43^,^44^. Therefore, we tested whether prestin can directly and specifically interact with cholesterol *in vitro*. Using cholesterol-coupled affinity beads^45^,^46^, we performed a pull-down assay with YFP-tagged prestin heterologously expressed in a stable HEK cell line^47^. Although a trace amount of prestin protein was pulled down by cholesterol-conjugated beads, the majority of prestin molecules did not bind to the cholesterol beads (Supplementary Fig. 3). We reasoned that prestin may have already been in a complex with cholesterol in the stable HEK cell line, making it difficult to interact with the cholesterol beads.

**Figure 4:**
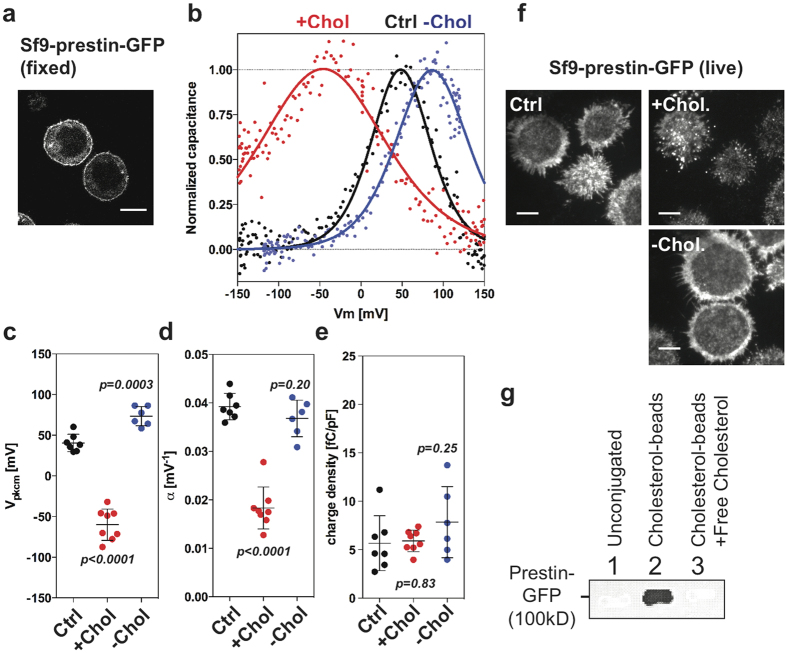
Interaction of prestin and cholesterol. (**a**) Representative images of Sf9 cells with prestin-GFP localizes at the plasma membrane. Scale bar, 10 μm. **(b–e**) Non-linear capacitance (NLC) measurements of stable Sf9-prestin-GFP cells. (**b**) Whole-cell recordings of Sf9-prestin-GFP cells were performed in control buffer (“Ctrl”), with cholesterol loading (“+Chol”), and with cholesterol depletion (“−Chol”). (**c**) Vpkcm. Mean ± s.d. are: Ctrl, 40 ± 11 mV (n = 7); +Chol, −60 ± 19 mV (n = 8); −Chol, 73 ± 12 mV (n = 6). P values are as indicated (t-test, compared to Ctrl). (**d**) alpha. Mean ± s.d. are: Ctrl, 0.039 ± 0.003 mV^−1^ (n = 7); +Chol, 0.018 ± 0.004 mV^−1^ (n = 8); −Chol, 0.037 ± 0.004 mV^−1^ (n = 6). P values are as indicated (t-test, compared to Ctrl). (**e**) Charge density. Mean ± s.d. are: Ctrl, 5.7 ± 2.8 fC/pF (n = 7); +Chol, 5.9 ± 1.1 fC/pF (n = 8); −Chol, 7.9 ± 3.7 fC/pF (n = 6). P values are as indicated (t-test, compared to Ctrl). (**f**) Live-cell TIRF imaging of Sf9-prestin-GFP cells with cholesterol manipulation. Sf9-prestin-GFP cells were either grown in control media (“Ctrl”), in media supplemented with 5 mM MβCD (“−Chol”), or 5 mM MβCD saturated with cholesterol (“Chol”) for 1 h and observed under TIRF microscopy. Scale bars, 5 μm. (**g**) Cholesterol pull-down assay of Sf9-prestin-GFP cells. Cell lysates containing membrane fractions from stable Sf9-prestin-GFP cells were incubated with unconjugated control beads or cholesterol-conjugated beads, separated on SDS-PAGE, and probed with anti-GFP. Prestin-GFP is pulled-down with cholesterol-conjugated beads but not with unconjugated beads (n = 5, *p* = 0.0059, t-test). Lanes 1, Unconjugated beads; 2, Cholesterol-conjugated beads; 3, Cholesterol-conjugated beads with addition of exogenous free cholesterol.

In order to better observe an association between cholesterol and prestin, we turned to an Sf9 insect cell line with known low-cholesterol content (10–20 times less) compared to mammalian cells^30^,^48^, and established a stable cell line that expresses GFP-tagged WT-prestin. The resulting sf9-prestin-GFP cells express prestin-GFP at the plasma membrane (Fig. 4a). Consistent with the presence of functional prestin molecules at this location, sf9-prestin-GFP cells exhibit nonlinear capacitance (NLC), a signature of prestin’s motor activity^49^,^50^. The NLC of sf9-prestin-GFP cells responded to cholesterol loading and depletion in qualitatively the same manner as previously reported for isolated OHCs and prestin-expressing HEK293 cells^21^,^22^ (Fig. 4b–e). V_pkcm_ is a parameter that describes the optimal voltage operating point of prestin while alpha represents prestin’s voltage sensitivity. Significant hyperpolarization of V_pkcm_ and reduction in alpha were observed under the cholesterol loading condition, suggesting that cholesterol affects the voltage operating point and sensitivity of prestin, respectively (Fig. 4c,d). Because of the low cholesterol content in Sf9 cells compared to other mammalian cell types, depolarization in V_pkcm_ in response to cholesterol depletion was minimal as expected (Fig. 4b,c). Similarly, cholesterol loading resulted in aggregation of prestin in Sf9-prestin-WT cells as previously described in HEK cells^21^, while cholesterol depletion did not affect the prestin’s distribution (Fig. 4f). Charge density, which correlates the density of the amount of functional prestin molecules at the PM, was not affected by acute cholesterol manipulations (Fig. 4e). Using Sf9-prestin-GFP cells, we also were able to recapitulate prestin’s interaction with cholesterol-beads in the pull-down assay. Prestin-GFP was pulled-down with cholesterol beads but not with unconjugated control beads as shown in Fig. 4g. Band intensities were measured and compared for control vs. cholesterol beads as in lanes 1 and 2, (Student’s t-test, *p* = 0.0059, n = 5). Addition of exogenous free cholesterol abolished prestin-GFP binding, indicating that this interaction is specific (Fig. 4g, lane 3). These data suggest a direct interaction of prestin and cholesterol *in vitro*.

### OHCs isolated from Prestin-KOs tolerate HPβCD treatment

**Figure 5:**
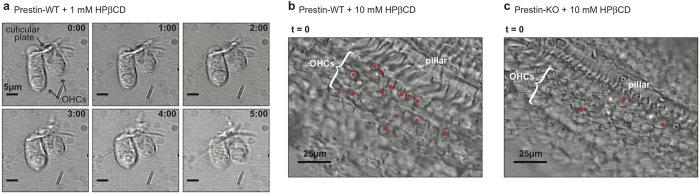
*In vitro* analysis of HPβCD-treated OHCs. (**a**) Time-lapse images of HPβCD-treated WT OHCs. Isolated OHCs from a WT-control mouse were treated with 1 mM HPβCD and observed under microscopy. OHCs rapidly shorten upon addition of HPβCD. Scale bars, 5 μm. (**b**) HPβCD treatment of an isolated OC from a WT mouse treated with 10 mM HPβCD. The initial image (t = 0, before the addition of HPβCD) is shown. Red asterisks (*) indicate cells that ruptured during the duration of time-lapse recording. (**c**) HPβCD treatment of an isolated OC from a prestin-KO mouse treated with 10 mM HPβCD. The initial image (t = 0, before the addition of HPβCD) is shown. Red asterisks (*) indicate cells that ruptured during the duration of time-lapse recording; white asterisks are OHCs that seem to have swollen but not ruptured.

Because of the close relationship between prestin and cholesterol, depletion of cholesterol may compromise the stability of the prestin-enriched LM, resulting in HPβCD-induced OHC death. In fact, Rajagopalan and colleagues noted “a drastic morphological change in OHCs and even cell death” while treating isolated OHCs with methyl-β-cyclodextrin (MβCD), which has a higher binding affinity to cholesterol than HPβCD^21^,^51^. We also observed rapid shortening of isolated WT OHCs and morphological changes in the OC upon addition of 1 mM HPβCD, a concentration commonly used in cell culture media (Fig. 5a and Supplemental Movie 1). At a higher HPβCD concentration (10 mM), we noticed OHC “rupture” in OC explants from WT animals where OHC nuclei seemed to suddenly (within one frame of a 4 sec interval) increase in size and then shrink (Fig. 5b, red asterisks and Supplemental Movie 2). Interestingly, the OHC “rupture” events were not evident in prestin-KO mice during a comparable duration of time-lapse recordings. Although there appeared to be some gradual increase in OHC size over time (in ~4 frames in a 4 sec interval, white asterisks), sudden “rupture” as seen in WT OCs was largely absent (Fig. 5c and Supplemental Movie 3). Thus, our *in vitro* observations corroborate the *in vivo* OHC survival data (see Fig. 1), i.e., prestin-KO OHCs tolerate the same HPβCD treatment that damages WT OHCs. These observations suggest that the extraction of cholesterol by HPβCD results in rapid membrane disruption of OHCs in a prestin-dependent manner, leading to cell death.

## Discussion

In this study, we identified prestin as a contributor to HPβ CD ototoxicity. Single, high-dose administration of HPβCD resulted in massive OHC loss in WT mice whereas OHCs were preserved in prestin-KO mice, indicating that prestin is involved (Fig. 1). Prestin localizes to the LM of OHCs, a specialized domain that undergoes voltage-dependent length and stiffness changes^16^,^28^,^52^. It is also known that the cholesterol content of the OHC’s LM is low when measured using filipin^20^,^21^,^32^ or TNM-FL (Fig. 3). Nevertheless, the LM must contain cholesterol as significant functional modulation of prestin can be seen in isolated OHCs using a cholesterol-depleting procedure^21^. Regardless of the abundance of cholesterol in the LM, it is clear that OHCs are sensitive to perturbation by HPβCD, as the toxic effect of HPβCD is rapid and limited to OHCs (Fig. 5a,b, and Supplemental Movies 1 and 2). Although our data show that prestin can interact with cholesterol *in vitro* (Fig. 4g), the identification of cholesterol binding motifs in prestin is required in order to determine whether disruption of the cholesterol-prestin interaction plays a role in OHC death. Yamashita *et al*. recently suggested that the OHC’s trilaminate structure, which consists of PM, actin-spectrin cortical lattice (CL), and the subsurface cisternae (SSC), limits prestin’s mobility^53^. It is also reported that salicylate, a prestin inhibitor that reversibly alters the SSC^54^, can increase prestin’s mobility in the presence MβCD^53^. Although the degree to which the OHC’s trilaminate structure depends on prestin is debatable^53^,^55^, it remains possible that the CL or SSC contribute to HPβCD-induced ototoxicity. Further investigation is required to fully elucidate how depletion of the cholesterol from the prestin-enriched LM induces cell death. The rapidity of this effect is consistent with a recent report^56^ showing that antioxidants do not protect OHCs against HPβCD toxicity, suggesting that cell death may not involve generation of ROS and apoptotic signaling events.

It is curious that the preservation of OHCs in HPβCD-treated prestin-KO cochleae was not statistically significant in the apical region but was limited to the high-frequency basal region (Fig. 1a,b), where NPC1 patients also experience impairment^57^. This observation indicates that factors in addition to prestin are likely involved, as cholesterol can influence a variety of biological processes by modulating various membrane proteins. Coincidentally, efferent nerve innervation is the highest in the middle region where OHC death occurred in both WT and prestin-KO cochleae^58^, raising the possibility that changes in the functions of additional proteins may underlie HPβCD ototoxicity. Efferent innervation to OHCs is mediated by Ca^2+^ entry through α9/α10-nicotinic receptors, followed by the activation of small-conductance (SK2) and the voltage- and Ca^2+^ -activated large conductance potassium (BK) channels^59^. Although the distribution of α9/α10-nicotinic receptors along the length of the cochlea is not clear, SK2 channels and BK channels are not evenly distributed, i.e., high expression is only found in apical and middle cochlear turns^59^. Intriguingly, both nicotinic acetylcholine receptors and BK channels are cholesterol-binding proteins, and their functions are regulated in part by cholesterol^2^. In addition, voltage-gated Ca^2+^ channels (VGCC) and BK channels in chicken hair cells are reported to be sensitive to cholesterol depletion by MβCD^60^, suggesting that increased influx of Ca^2+^ could result in excitotoxicity and cell death. Although it is not clear whether innervation densities or channel distributions are altered in prestin-KO mice, it is possible that these factors contribute to OHC loss in the apical half of prestin-KO cochleae in response to cholesterol extraction by HPβCD.

HPβCD is the only promising drug for ameliorating the neurodegenerative phenotype in NPC1 patients. It is, therefore, important to elucidate mechanisms of ototoxicity to avoid further hearing impairment in afflicted individuals. Recent attempts in minimizing adverse effects of β-CDs include specific targeting of HPβCD to lysosomes by tethering the cyclodextrins to biocleavable polyrotaxanes (HE-SS-PRX)^61^. Although *in vitro* studies show reduced cytotoxicity by preventing β-CDs from targeting the plasma membrane, it is concerning that cellular access of HE-SS-PRX depends on uptake via endocytic pathways. It, therefore, remains to be seen whether HE-SS-PRX can maintain its efficacy without causing toxicity to OHCs *in vivo*. These complications relate to the fact that HPβCD does not cross the blood-brain barrier^62^, with the result that current treatment involves direct administration to CSF. Since OHCs are bathed in perilymph, which is derived from CSF, it may be difficult to avoid damage to OHCs with the current drug-delivering strategies. Additional study is required before we are able to develop improved HPβCD-based therapies to minimize ototoxicity in NPC1 patients.

## Methods

### Animals

All experimental procedures were conducted in accordance with the Guide for the Care and Use of Laboratory Animals by NIH, and were approved by Northwestern University’s Animal Care and Use Committee. Details on the generation and characterization of prestin knockout (KO) mice is described elsewhere^28^. In order to minimize OHC death in mice lacking prestin^38^, the prestin KO mice were backcrossed to the FVB strain for 8 generations (N8). This strain is known to have excellent high-frequency hearing well into adulthood^63^. Prestin wild-type (WT) and KO mice on the FVB background were obtained by Het x Het breeding.

### HPβCD treatment and *in vivo* physiology

After weaning WT and prestin-KO mice were injected with 0.9% NaCl or 8000 mg/kg HPβCD dissolved in 0.9% NaCl (Sigma, H107) subcutaneously as described previously^10^. Distortion product otoacoustic emissions (DPOAEs) were measured by presenting the two stimulating primaries each at 70 dB SPL and for a frequency ratio of f2/f1 = 1.2. Auditory brainstem responses (ABR) were also collected to document the output of the cochlea, as well as subsequent neural processing in the brainstem. For these recordings, subcutaneous electrodes were inserted at the vertex and the mastoid with each response measured relative to the indifferent electrode inserted at the opposite shoulder/neck region. Calibration was performed quasi-free field as reported previously^64^. Thresholds were determined by noting the signal level where the ABR waveform disappeared into the noise. Because the number of averages increased as signal level decreased (5 dB step size), the noise level was ~0.2 microvolts. Further details on how these recordings are made appear in a previous publication^65^.

### Tissue processing and immunostaining

Mice were cardiac perfused with 4% paraformaldehyde and cochleae extracted at 7 days post treatment unless otherwise noted. After post-fixation and decalcification, cochleae were dissected following the Eaton-Peabody Laboratory cochlear dissection protocol^66^. For immunostaining, anti-prestin^67^ and anti-oncomodulin (Santa Cruz) antibodies were used. Secondary antibodies include goat anti-rabbit Alexa Fluor 488 and donkey anti-goat Alexa Fluor 488 (Molecular Probes). For cholesterol staining, cochlear sections were incubated with 1 μM TNM-FL^33^ for 3 days at 4 °C without permeabilization. Stained cochlear sections were mounted onto slides using Dako fluorescent mounting medium (DAKO).

### Imaging and anatomical measurements

Images were captured on a Nikon C2+ or A1R confocal microscope with Plan Fluor 10X and Plan Apo 20X objectives (Nikon) controlled by NIS Element software. For TNM imaging, a Plan Apo 60X oil objective (Nikon) was used. Z-stack images were taken with 0.4 μm intervals and 3D images reconstructed using NIS Element software. Basilar membrane length was measured using ImageJ, and the numbers of remaining OHCs determined. A mouse cochlear place-frequency map^68^ was used to determine the corresponding frequencies. An Andor XDI Revolution microscope with Apo TIRF 100X oil objective (Nikon) was used for TIRF imaging.

### Plasmids

To generate pIZ-gPres-ceGFP, WT gerbil prestin (gPrestin) with C-terminal EGFP tag was amplified from gPrestin-GFP^69^, and cloned into a pIZ/V5-His vector (Invitrogen) using SpeI and XhoI.

### Cell Line and cell culture

Sf9 cells (Invitrogen) were maintained in Sf-900 III SFM supplemented with 5% fetal bovine serum (Gibco) and 1X antibiotic antimycotic solution (Sigma). To generate stable Sf9 cells with WT gPrestin, Sf9 cells were transfected with pIZ-gPres-ceGFP using Effectene (Qiagen), and selected with 1 μg/μl zeocin (Invitrogen). A single clone was chosen to establish the stable cell line. The tetracycline-inducible WT-gPrestin stable HEK293 cell line (293-TRxST-gPrestin-YFP4TOmycHisC) was a generous gift from Drs. Santo-Sacchi and Navaratnam. Growth conditions and induction of the cell line were defined previously^47^. For transient transfections of Sf9 and HEK293T cells with prestin constructs, Effectene (Qiagen) and jetPRIME (Polyplus) were used according to the manufacture’s instructions.

### Nonlinear capacitance (NLC) measurements

Whole-cell recordings were performed at room temperature using the Axopatch 200 A amplifier (Molecular Devices, CA). Recording pipettes were pulled from borosilicate glass to achieve initial bath resistances averaging ~3 MΩ. Intracellular pressure was kept at 0 mmHg during recording. Recording pipettes were filled with an intracellular solution containing (mM): 140 CsCl, 2 MgCl_2_, 10 EGTA, and 10 HEPES (pH 7.3). Cells were bathed in an extracellular solution containing (mM): 120 NaCl, 20 TEA-Cl, 2 CoCl_2_, 2 MgCl_2_, 10 HEPES (pH 7.3). Osmolarity was adjusted to 310 mmol/kg with glucose. NLC was measured using a sinusoidal voltage stimulus (2.5-Hz, 120 or 150 mV amplitude) superimposed with two higher frequency stimuli (391 and 781 Hz, 10 mV amplitude)^70^. Data were collected by jClamp (SciSoft Company, New Haven, CT), and NLC determined as described previously^70^. For cholesterol loading and depletion, 5 mM MβCD was used with or without a saturating amount of cholesterol.

Voltage-dependent cell membrane electric capacitance data were analyzed using the following two-state Boltzmann equation:


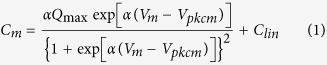


where α is the slope factor of the voltage-dependence of charge transfer, Q_max_ is the maximum charge transfer, V_m_ is the membrane potential, V_pkcm_ is the voltage at which the maximum charge movement is attained, and C_lin_ is the linear capacitance^70^.

### Preparation of Sf9 cell lysates

Sf9 cells stably expressing WT-gPrestin were collected and washed once in PBS. Cell pellets were first resuspended in wash buffer (50 mM Tris-Cl buffer (pH 7.4), 1X protease inhibitor cocktail (Sigma, P8849) and 1 mM PMSF (Sigma, P7626)), and incubated on ice for 30 min. Cells were then homogenized, and spun down at 700 g for 10 min at 4 °C. Supernatants were then transferred to a fresh tube, and spun down at 10,000 g for 30 min at 4 °C. The total membrane fraction of Sf9 cells (pellets) were dissolved in extraction buffer (50 mM Tris-Cl, pH 7.4, 50 mM NaCl, 25 mM n-dodecyl β-d-maltoside (DDM), 1 mM EGTA, 1X protease inhibitor cocktail, and 1 mM PMSF) at room temperature for 1 h, and centrifuged at 16,000 g for 30 min at room temperature. Supernatants containing gPrestin-GFP were used for the cholesterol binding assay.

### Cholesterol binding assay

Preparation of cholesterol beads was based on a previous report^45^,^46^ and the manufacturer’s instructions. CarboxyLink coupling gel (Thermo Scientific) was washed in 30% 1,4-dioxane (Fisher) and then in 100% dioxane. Cholesteryl hemisuccinate (Sigma, C6512) was dissolved in 100% dioxane, and immobilized on a pre-washed CarboxyLink coupling gel in the presence of N,N’-dicyclohexylcarbodiimide (DCC; Thermo Scientific, 20320) at room temperature for 4 hours. The cholesterol-beads were then washed twice each in 100% dioxane, 30% dioxane, and then stored in PBS. At the same time, pre-washed CarboxyLink coupling gel was processed without cholesteryl hemisuccinate to serve as a control for non-specific binding. Cholesterol-beads or unconjugated control beads were mixed with cell lysates containing membrane fractions isolated from stable Sf9 cells (described above) in the presence or absence of 1 mM cholesterol, and incubated for 1 h at room temperature. The reaction mix was then centrifuged and washed 5 times with 50 mM Tris, and eluted with 2X Laemmli Sample Buffer (BioRad) at room temperature for 30 min. Eluates were analyzed by Western blotting.

### Western blotting

For detection of gPrestin expressed in Sf9 cells, samples were separated on 4–20% gradient Mini-Protein precast gels (BioRad), transferred to 0.45 μm nitrocellulose membranes (BioRad) and probed with 1:5000 dilutions of anti-V5 antibodies (Invitrogen) followed by 1:5000 dilutions of goat anti-mouse-HRP antibodies (Jackson Laboratory). For detection of gPrestin expressed in HEK293T cells, anti-GFP (Rockland) and anti-chicken-HRP (Jackson Laboratory) antibodies were used.

## Additional Information

**How to cite this article**: Takahashi, S. *et al*. Susceptibility of outer hair cells to cholesterol chelator 2- hydroxypropyl-β-cyclodextrine is prestin-dependent. *Sci. Rep.* **6**, 21973; doi: 10.1038/srep21973 (2016).

## Supplementary Material

Supplementary Information

Supplementary Information

Supplementary Information

Supplementary Information

## References

[b1] Janmey P. A. & Kinnunen P. K. J. Biophysical properties of lipids and dynamic membranes. Trends Cell Biol. 16, 538–546, doi: 10.1016/j.tcb.2006.08.009 (2006). 16962778

[b2] Levitan I., Singh D. K. & Rosenhouse-Dantsker A. Cholesterol binding to ion channels. Front. Physiol. 5, doi: 10.3389/fphys.2014.00065 (2014). PMC3935357 24616704

[b3] Simons K. & Toomre D. Lipid rafts and signal transduction. Nat. Rev. Mol. Cell Biol. 1, 31–39, doi: 10.1038/35036052 (2000). 11413487

[b4] Gimpl G. Cholesterol-protein interaction: methods and cholesterol reporter molecules. In Cholesterol Binding and Cholesterol Transport Proteins. Vol. 51, (ed. Harris J. R.) 1–45 (Springer Netherlands, 2010). 10.1007/978-90-481-8622-8_1 20213539

[b5] Loftsson T. & Duchene D. Cyclodextrins and their pharmaceutical applications. Int. J. Pharm. 329, 1–11, doi: 10.1016/j.ijpharm.2006.10.044 (2007). 17137734

[b6] Abi-Mosleh L., Infante R. E., Radhakrishnan A., Goldstein J. L. & Brown M. S. Cyclodextrin overcomes deficient lysosome-to-endoplasmic reticulum transport of cholesterol in Niemann-Pick type C cells. Proc. Natl. Acad. Sci. USA 106, 19316–19321, doi: 10.1073/pnas.0910916106 (2009). 19884502 PMC2780767

[b7] Vance J. E. Lipid imbalance in the neurological disorder, Niemann-Pick C disease. FEBS Lett. 580, 5518–5524, doi: 10.1016/j.febslet.2006.06.008 (2006). 16797010

[b8] Aqul A. . Unesterified cholesterol accumulation in late endosomes/lysosomes causes neurodegeneration and is prevented by driving cholesterol export from this compartment. J. Neurosci. 31, 9404–9413, doi: 10.1523/JNEUROSCI.1317-11.2011 (2011). 21697390 PMC3134878

[b9] Camargo F. . Cyclodextrins in the treatment of a mouse model of Niemann-Pick C disease. Life Sci. 70, 131–142 (2001). 11787939 10.1016/s0024-3205(01)01384-4

[b10] Crumling M. A. . Hearing loss and hair cell death in mice given the cholesterol-chelating agent hydroxypropyl-β-cyclodextrin. PLoS One 7, e53280, doi: 10.1371/journal.pone.0053280 (2012). 23285273 PMC3532434

[b11] King K. . HPβCD therapy in humans with NPC1 disease: Audiological Outcomes. 38th meeting of the Association for Research in Otolaryngology, 297, Baltimore, MD (2015).

[b12] King K. A. . Auditory phenotype of Niemann-Pick disease, type C1. Ear Hear. 35, 110–117, doi: 10.1097/AUD.0b013e3182a362b8 (2014). 24225652 PMC3895917

[b13] Cronin S., Lin A., Thompson K., Hoenerhoff M. & Duncan R. K. Hearing Loss and Otopathology Following Systemic and Intracerebroventricular Delivery of 2-Hydroxypropyl-Beta-Cyclodextrin. J Assoc Res Otolaryngol 16, 599–611, doi: 10.1007/s10162-015-0528-6 (2015). 26055150 PMC4569609

[b14] Ashmore J. Cochlear outer hair cell motility. Physiol. Rev. 88, 173–210, doi: 10.1152/physrev.00044.2006 (2008). 18195086

[b15] Dallos P. . Prestin-based outer hair cell motility is necessary for mammalian cochlear amplification. Neuron 58, 333–339, doi: 10.1016/j.neuron.2008.02.028 (2008). 18466744 PMC2435065

[b16] Zheng J. . Prestin is the motor protein of cochlear outer hair cells. Nature 405, 149–155, doi: 10.1038/35012009 (2000). 10821263

[b17] Alper S. L. & Sharma A. K. The SLC26 gene family of anion transporters and channels. Mol. Aspects Med. 34, 494–515, doi: 10.1016/j.mam.2012.07.009 (2013). 23506885 PMC3602804

[b18] Hallworth R. & Nichols M. G. Prestin in HEK cells is an obligate tetramer. J. Neurophysiol. 107, 5–11, doi: 10.1152/jn.00728.2011 (2012). 21975444 PMC3349699

[b19] Zheng J. . Analysis of the oligomeric structure of the motor protein prestin. J. Biol. Chem. 281, 19916–19924, doi: 10.1074/jbc.M513854200 (2006). 16682411

[b20] Brownell W. E., Jacob S., Hakizimana P., Ulfendahl M. & Fridberger A. Membrane cholesterol modulates cochlear electromechanics. Pflugers Arch. 461, 677–686, doi: 10.1007/s00424-011-0942-5 (2011). 21373862 PMC3098987

[b21] Rajagopalan L. . Tuning of the outer hair cell motor by membrane cholesterol. J. Biol. Chem. 282, 36659–36670, doi: 10.1074/jbc.M705078200 (2007). 17933870 PMC2679373

[b22] Sfondouris J., Rajagopalan L., Pereira F. A. & Brownell W. E. Membrane composition modulates prestin-associated charge movement. J. Biol. Chem. 283, 22473–22481, doi: 10.1074/jbc.M803722200 (2008). 18567583 PMC2504877

[b23] Fang J., Izumi C. & Iwasa K. H. Sensitivity of prestin-based membrane motor to membrane thickness. Biophys. J. 98, 2831–2838, doi: 10.1016/j.bpj.2010.03.034 (2010). 20550895 PMC2884244

[b24] Iwasa K. H. Effect of stress on the membrane capacitance of the auditory outer hair cell. Biophys. J. 65, 492–498, doi: 10.1016/S0006-3495(93)81053-1 (1993). 8369452 PMC1225741

[b25] Kakehata S. & Santos-Sacchi J. Membrane tension directly shifts voltage dependence of outer hair cell motility and associated gating charge. Biophys. J. 68, 2190–2197, doi: 10.1016/S0006-3495(95)80401-7 (1995). 7612863 PMC1282124

[b26] Santos-Sacchi J. & Huang G. Temperature dependence of outer hair cell nonlinear capacitance. Hear. Res. 116, 99–106 (1998). 9508032 10.1016/s0378-5955(97)00204-9

[b27] Nilsen N., Brownell W. E., Sun S. X. & Spector A. A. Effect of membrane mechanics on charge transfer by the membrane protein prestin. Biomech Model Mechanobiol 11, 107–118, doi: 10.1007/s10237-011-0296-0 (2012). 21365198 PMC3158267

[b28] Liberman M. C. . Prestin is required for electromotility of the outer hair cell and for the cochlear amplifier. Nature 419, 300–304, doi: 10.1038/nature01059 (2002). 12239568

[b29] Cheatham M. A., Huynh K. H., Gao J., Zuo J. & Dallos P. Cochlear function in Prestin knockout mice. J. Physiol. 560, 821–830, doi: 10.1113/jphysiol.2004.069559 (2004). 15319415 PMC1665294

[b30] Gimpl G., Klein U., Reiländer H. & Fahrenholz F. Expression of the human oxytocin receptor in baculovirus-infected insect cells: high-affinity binding is induced by a cholesterol-cyclodextrin complex. Biochemistry 34, 13794–13801 (1995). 7577972 10.1021/bi00042a010

[b31] Kilsdonk E. P. . Cellular cholesterol efflux mediated by cyclodextrins. J. Biol. Chem. 270, 17250–17256 (1995). 7615524 10.1074/jbc.270.29.17250

[b32] Nguyen T. V. & Brownell W. E. Contribution of membrane cholesterol to outer hair cell lateral wall stiffness. Otolaryngol. Head Neck Surg. 119, 14–20 (1998). 9674509 10.1016/S0194-5998(98)70167-6

[b33] Nishimura S. . Marine antifungal theonellamides target 3β-hydroxysterol to activate Rho1 signaling. Nat. Chem. Biol. 6, 519–526, doi: 10.1038/nchembio.387 (2010). 20543850

[b34] Espiritu R. A. . Interaction between the Marine Sponge Cyclic Peptide Theonellamide A and Sterols in Lipid Bilayers As Viewed by Surface Plasmon Resonance and Solid-State 2H Nuclear Magnetic Resonance. Biochemistry 52, 2410–2418, doi: 10.1021/bi4000854 (2013). 23477347

[b35] Nishimura S. . Visualization of Sterol-Rich Membrane Domains with Fluorescently-Labeled Theonellamides. PLoS One 8, e83716, doi: 10.1371/journal.pone.0083716 (2013). 24386262 PMC3873978

[b36] Cheatham M. A. . Evaluation of an independent prestin mouse model derived from the 129S1 strain. Audiology &amp; neuro-otology 12, 378–390, doi: 10.1159/000106481 (2007). 17664869

[b37] Cheatham M. A. . Prestin-Dependence of Outer Hair Cell Survival and Partial Rescue of Outer Hair Cell Loss in PrestinV499G/Y501H Knockin Mice. PLoS One 10, e0145428, doi: 10.1371/journal.pone.0145428 (2015). 26682723 PMC4684303

[b38] Wu X., Gao J., Guo Y. & Zuo J. Hearing threshold elevation precedes hair-cell loss in prestin knockout mice. Brain research. Molecular brain research 126, 30–37, doi: 10.1016/j.molbrainres.2004.03.020 (2004). 15207913

[b39] Rajagopalan L. . Glycosylation regulates prestin cellular activity. J. Assoc. Res. Otolaryngol. 11, 39–51, doi: 10.1007/s10162-009-0196-5 (2010). 19898896 PMC2820205

[b40] Sturm A. K., Rajagopalan L., Yoo D., Brownell W. E. & Pereira F. A. Functional expression and microdomain localization of prestin in cultured cells. Otolaryngol. Head Neck Surg. 136, 434–439, doi: 10.1016/j.otohns.2006.10.030 (2007). 17321873 PMC2679365

[b41] Canis M. . The influence of cholesterol on the motility of cochlear outer hair cells and the motor protein prestin. Acta Otolaryngol. 129, 929–934, doi: 10.1080/00016480802495438 (2009). 19153847

[b42] Pelletier R. M. & Vitale M. L. Filipin vs enzymatic localization of cholesterol in guinea pig, mink, and mallard duck testicular cells. J. Histochem. Cytochem. 42, 1539–1554, doi: 10.1177/42.12.7983355 (1994). 7983355

[b43] Severs N. J. & Simons H. L. Failure of filipin to detect cholesterol-rich domains in smooth muscle plasma membrane. Nature 303, 637–638 (1983). 6855909 10.1038/303637a0

[b44] Steer C. J., Bisher M., Blumenthal R. & Steven A. C. Detection of membrane cholesterol by filipin in isolated rat liver coated vesicles is dependent upon removal of the clathrin coat. J. Cell Biol. 99, 315–319, doi: 10.1083/jcb.99.1.315 (1984). 6145719 PMC2275625

[b45] Wichman A. Affinity chromatography of human plasma low- and high-density lipoproteins. Elution by selective cleavage of a bond in the spacer. The Biochemical journal 181, 691–698 (1979). 229822 10.1042/bj1810691 PMC1161209

[b46] Schroeder C., Heider H., Moncke-Buchner E. & Lin T. I. The influenza virus ion channel and maturation cofactor M2 is a cholesterol-binding protein. Eur. Biophys. J. 34, 52–66, doi: 10.1007/s00249-004-0424-1 (2005). 15221235

[b47] Bian S., Koo B. W., Kelleher S., Santos-Sacchi J. & Navaratnam D. S. A highly expressing Tet-inducible cell line recapitulates *in situ* developmental changes in prestin’s Boltzmann characteristics and reveals early maturational events. Am. J. Physiol. Cell physiol. 299, C828–835, doi: 10.1152/ajpcell.00182.2010 (2010). 20631244 PMC3774197

[b48] Marheineke K., Grünewald S., Christie W. & Reiländer H. Lipid composition of Spodoptera frugiperda (Sf9) and Trichoplusia ni (Tn) insect cells used for baculovirus infection. FEBS Lett. 441, 49–52 (1998). 9877163 10.1016/s0014-5793(98)01523-3

[b49] Ashmore J. F. Forward and reverse transduction in the mammalian cochlea. Neurosci. Res. Suppl. 12, S39–50 (1990). 2243636 10.1016/0921-8696(90)90007-p

[b50] Santos-Sacchi J. Reversible inhibition of voltage-dependent outer hair cell motility and capacitance. J. Neurosci. 11, 3096–3110 (1991). 1941076 10.1523/JNEUROSCI.11-10-03096.1991 PMC6575435

[b51] Irie T. & Uekama K. Pharmaceutical applications of cyclodextrins. III. Toxicological issues and safety evaluation. J. Pharm. Sci. 86, 147–162, doi: 10.1021/js960213f (1997). 9040088

[b52] He D. Z. Z., Jia S. & Dallos P. Prestin and the dynamic stiffness of cochlear outer hair cells. J. Neurosci. 23, 9089–9096 (2003). 14534242 10.1523/JNEUROSCI.23-27-09089.2003 PMC6740818

[b53] Yamashita T. . Outer Hair Cell Lateral Wall Structure Constrains the Mobility of Plasma Membrane Proteins. PLoS Genet. 11, e1005500, doi: 10.1371/journal.pgen.1005500 (2015). 26352669 PMC4564264

[b54] Dieler R., Shehata-Dieler W. E. & Brownell W. E. Concomitant salicylate-induced alterations of outer hair cell subsurface cisternae and electromotility. J. Neurocytol. 20, 637–653 (1991). 1940979 10.1007/BF01187066

[b55] He D. Z. . Changes in plasma membrane structure and electromotile properties in prestin deficient outer hair cells. Cytoskeleton (Hoboken) 67, 43–55, doi: 10.1002/cm.20423 (2010). 20169529 PMC2842980

[b56] Kim Y. H. . Ototoxicity of 2-hydroxypropyl-β-cyclodextrin (HPβCD) in mice. 38th meeting of the Association for Research in Otolaryngology, 795, Baltimore, MD (2015).

[b57] Ottinger E. A. . Collaborative development of 2-hydroxypropyl-β-cyclodextrin for the treatment of Niemann-Pick type C1 disease. Curr. Top. Med. Chem. 14, 330–339 (2014). 24283970 10.2174/1568026613666131127160118 PMC4048128

[b58] Maison S. F., Adams J. C. & Liberman M. C. Olivocochlear innervation in the mouse: immunocytochemical maps, crossed versus uncrossed contributions, and transmitter colocalization. J. Comp. Neurol. 455, 406–416, doi: 10.1002/cne.10490 (2003). 12483691 PMC1805785

[b59] Maison S. F., Pyott S. J., Meredith A. L. & Liberman M. C. Olivocochlear suppression of outer hair cells *in vivo*: evidence for combined action of BK and SK2 channels throughout the cochlea. J. Neurophysiol. 109, 1525–1534, doi: 10.1152/jn.00924.2012 (2013). 23282326 PMC3602942

[b60] Purcell E. K., Liu L., Thomas P. V. & Duncan R. K. Cholesterol influences voltage-gated calcium channels and BK-type potassium channels in auditory hair cells. PLoS One 6, e26289, doi: 10.1371/journal.pone.0026289 (2011). 22046269 PMC3194812

[b61] Tamura A. & Yui N. Lysosomal-specific cholesterol reduction by biocleavable polyrotaxanes for ameliorating Niemann-Pick type C disease. Sci. Rep. 4, 4356, doi: 10.1038/srep04356 (2014). 24619155 PMC3950578

[b62] Pontikis C. C., Davidson C. D., Walkley S. U., Platt F. M. & Begley D. J. Cyclodextrin alleviates neuronal storage of cholesterol in Niemann-Pick C disease without evidence of detectable blood-brain barrier permeability. J. Inherited Metab. Dis. 36, 491–498, doi: 10.1007/s10545-012-9583-x (2013). 23412751 PMC3929395

[b63] Zheng Q. Y., Johnson K. R. & Erway L. C. Assessment of hearing in 80 inbred strains of mice by ABR threshold analyses. Hearing Res. 130, 94–107, doi: 10.1016/S0378-5955(99)00003-9 (1999). PMC2855304 10320101

[b64] Pearce M., Richter C. P. & Cheatham M. A. A reconsideration of sound calibration in the mouse. J. Neurosci. Methods 106, 57–67 (2001). 11248341 10.1016/s0165-0270(01)00329-6

[b65] Cheatham M. A. . Loss of the tectorial membrane protein CEACAM16 enhances spontaneous, stimulus-frequency, and transiently evoked otoacoustic emissions. J. Neurosci. 34, 10325–10338, doi: 10.1523/JNEUROSCI.1256-14.2014 (2014). 25080593 PMC4115139

[b66] Liberman L. D. & Liberman M. C. Dynamics of cochlear synaptopathy after acoustic overexposure. J. Assoc. Res. Otolaryngol. 16, 205–219, doi: 10.1007/s10162-015-0510-3 (2015). 25676132 PMC4368657

[b67] Zheng J. . The C-terminus of prestin influences nonlinear capacitance and plasma membrane targeting. J. Cell Sci. 118, 2987–2996, doi: 10.1242/jcs.02431 (2005). 15976456

[b68] Müller M., von Hünerbein K., Hoidis S. & Smolders J. W. T. A physiological place-frequency map of the cochlea in the CBA/J mouse. Hearing Res. 202, 63–73, doi: 10.1016/j.heares.2004.08.011 (2005). 15811700

[b69] Matsuda K. . N-linked glycosylation sites of the motor protein prestin: effects on membrane targeting and electrophysiological function. J. Neurochem. 89, 928–938, doi: 10.1111/j.1471-4159.2004.02377.x (2004). 15140192

[b70] Homma K. & Dallos P. Evidence that prestin has at least two voltage-dependent steps. J. Biol. Chem. 286, 2297–2307, doi: 10.1074/jbc.M110.185694 (2011). 21071769 PMC3023524

