# Supplementary material for: Susceptibility of outer hair cells to cholesterol chelator 2-hydroxypropyl-β-cyclodextrine is prestin-dependent
Source: Sci Rep. 2016 Feb 23;6:21973. doi: 10.1038/srep21973 (PMC4763217; doi:10.1038/srep21973)
Supplement: Supplementary Information [file srep21973-s4.pdf]

Supplementary Information

Susceptibility of outer hair cells to cholesterol chelator 2-hydroxypropyl-β-cyclodextrine is prestin-dependent

Satoe Takahashi, Kazuaki Homma, Yingjie Zhou, Shinichi Nishimura, Chongwen Duan, Jessie Chen, Aisha Ahmad, Mary Ann Cheatham, and Jing Zheng

Corresponding author: Jing Zheng
Department of Otolaryngology – Head and Neck Surgery
Feinberg School of Medicine, Northwestern University
320 East Superior Street
Searle 12-573
Chicago, IL 60611
jzh215@northwestern.edu
(312)-503-3417

Prestin-KO (Mid-Base)

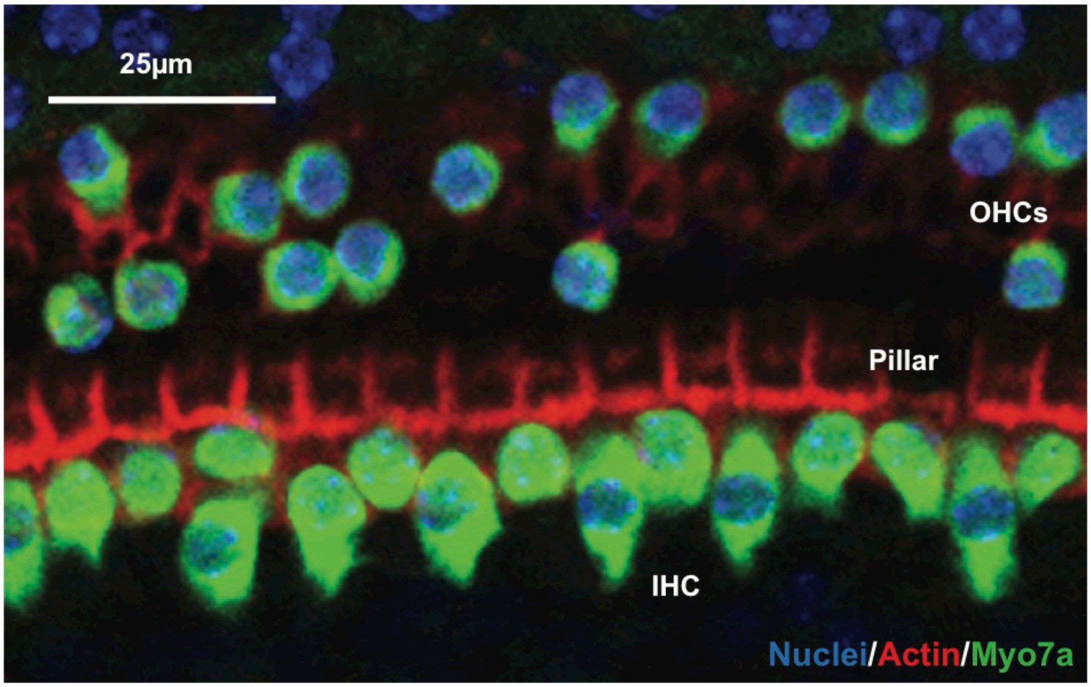

Supplemental Fig. 1. Only OHCs are damaged by HPβCD treatment.

Representative image of HPβCD-treated cochlea from prestin-KO mice, stained with anti-Myosin 7a for IHCs and OHCs, Phalloidin-Alexa 546 for actin, and Hoechst 33342 for nuclei. All the IHCs and pillar cells remained intact while some OHC loss occurred in the mid-to-basal region. Scale bar, 25μm.

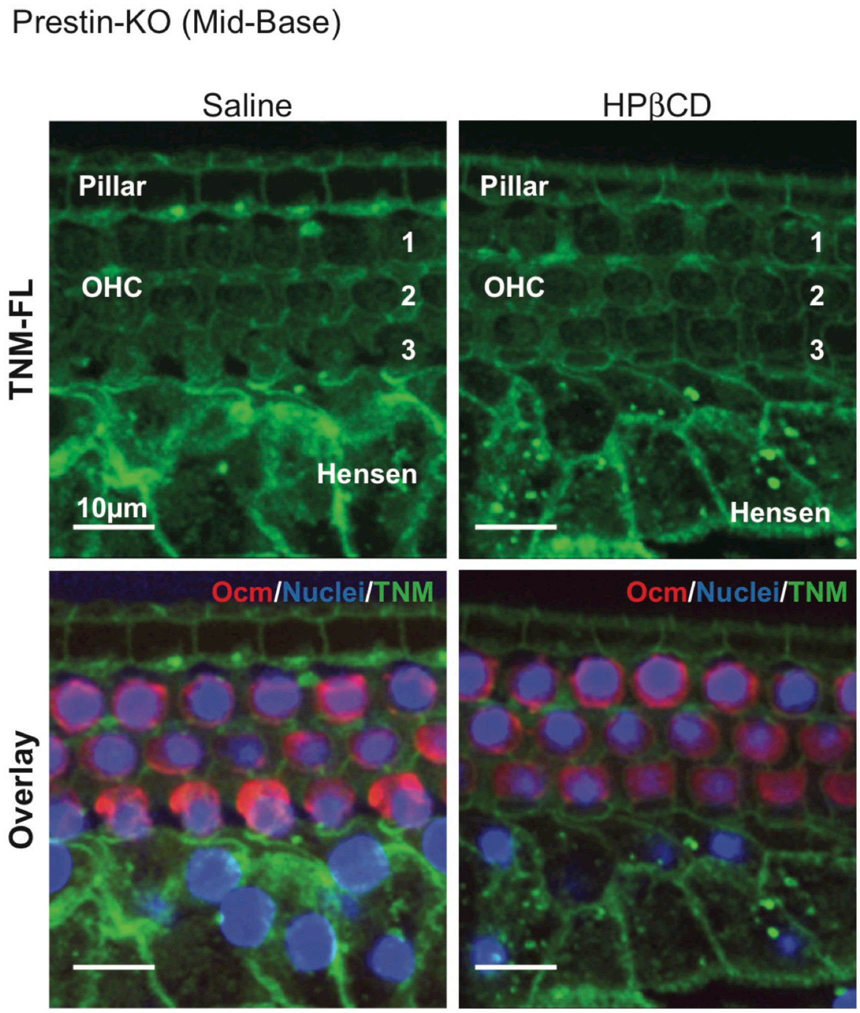

Supplemental Fig. 2. Cholesterol staining of prestin-KO cochleae.

Comparison of saline- and HPβCD-treated prestin-KO cochleae at the mid-to-basal region. An HPβCD-treated OC exhibits similar TNM-FL staining intensities as in the saline control. Red, anti-oncomodulin; Green, TNM-FL; Blue, Hoechst 33342 for nuclei. Scale bars, 5µm.

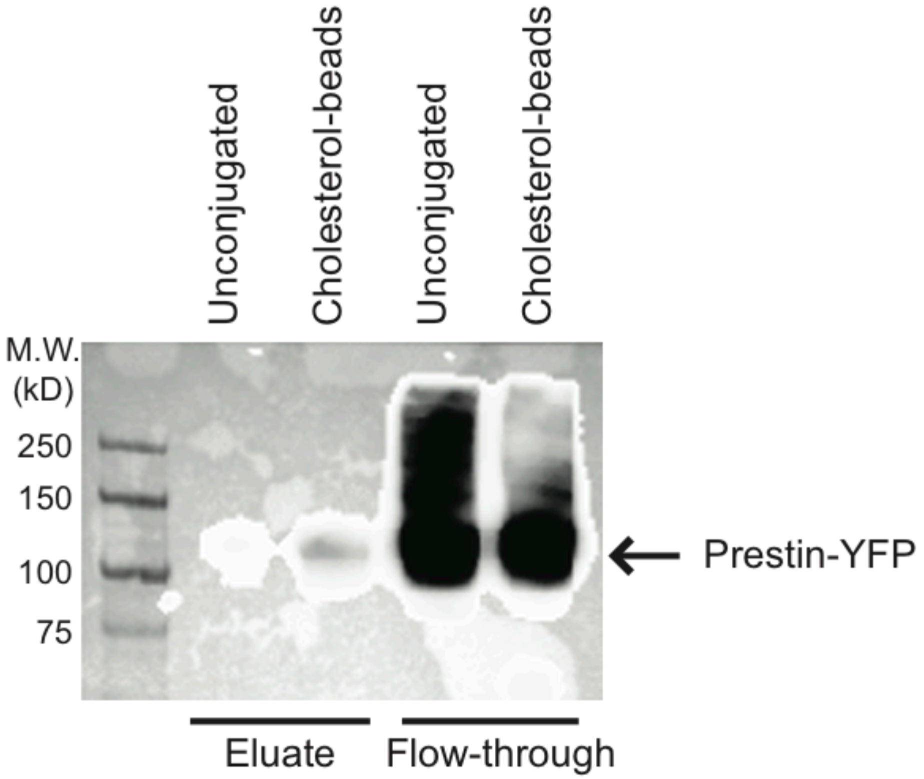

Supplemental Fig. 3. Pull-down assays using stable HEK293-prestin-YFP cells. Cell lysates containing membrane fractions were prepared from HEK293 cells stably expressing prestin-YFP, and incubated with unconjugated control beads or cholesterol-conjugated resins, separated on SDS-PAGE and probed with anti-GFP antibody. Prestin-YFP was pulled-down with cholesterol-beads (Lane 2) but not with unconjugated control beads (Lane 1). The flow-through data (Lanes 3 and 4) showed that the unbound prestin-YFP in both unconjugated control and cholesterol-beads conditions.

Supplemental Movie 1. Time-lapse images of HPβCD-treated WT OHCs. OHCs from prestin-WT were isolated and placed on a glass-bottom 35mm dish. HPβCD was added to the final concentration of 1 mM at t=0 (0:00), and imaged at 4 sec intervals for over 5 min. Scale bar, 5μm.

Supplemental Movie 2. Time-lapse images of HPβCD-treated WT OC. Sense organs from WT mice were dissected and placed on a glass-bottom 35mm dish. HPβCD was added to the final concentration of 10 mM at t=0 (0:00), and imaged at 4 sec intervals for over 12 min. Scale bar, 25μm.

Supplemental Movie 3. Time-lapse images of HPβCD-treated prestin-KO OC. Sense organs from prestin-KOs were dissected and placed on a glass-bottom 35mm dish. HPβCD was added to the final concentration of 10 mM at t=0 (0:00), and imaged at 4 sec intervals for over 12 min. Scale bar, 25μm.
